# Electronics‐Free Visual Monitoring of Dry Eye Disease‐Related Tear Biomarkers Using Smart Scleral Lenses

**DOI:** 10.1002/advs.77399

**Published:** 2026-08-27

**Authors:** Ziheng Wang, Sang Mok Park, Dawn Meyer, Seul Ah Lee, Yumin Dai, Tianhao Yu, Taewoong Park, Feiyang Li, Jason Gerard Jedlicka, Tristan Michael Long, Oluwabunmi T. Oladele, Gillian C. Shaw, Shin Ae Park, Pete S. Kollbaum, Young L. Kim, Chi Hwan Lee

**Affiliations:** ^1^ School of Mechanical Engineering Purdue University West Lafayette Indiana USA; ^2^ Department of Intelligent Systems Engineering Indiana University Bloomington Indiana USA; ^3^ School of Optometry Indiana University Bloomington Indiana USA; ^4^ Weldon School of Biomedical Engineering Purdue University West Lafayette Indiana USA; ^5^ School of Materials Engineering Purdue University West Lafayette Indiana USA; ^6^ Department of Veterinary Clinical Sciences Purdue University West Lafayette Indiana USA; ^7^ School of Veterinary Medicine University of Wisconsin‐Madison Madison Wisconsin USA; ^8^ Elmore Family School of Electrical and Computer Engineering Purdue University West Lafayette Indiana USA

**Keywords:** dry eye disease, ocular healthcare, scleral lenses, tear biomarkers, visual monitoring, wearable sensors

## Abstract

Dry eye disease (DED) is a prevalent ocular condition characterized by tear film instability, hyperosmolarity, inflammation, and neurosensory abnormalities. Its episodic and fluctuating nature complicates diagnosis and management, as conventional clinical assessments often fail to capture transient tear film changes. Although emerging tear‐sensing technologies show promise, many rely on trained personnel, only sporadic measurements, external readers, or integrated electronics, limiting their practicality for in‐eye applications. Here, we present an electronics‐free scleral lens platform for wearable monitoring of DED‐related tear biomarker changes using passive colorimetric sensors integrated within the lens tear reservoir. This configuration enables visual and smartphone‐assisted readout of chloride ions, pH, and ocular surface temperature within physiologically relevant ranges while avoiding direct contact with the corneal surface. The vaulted scleral lens geometry forms a stable, self‐replenishing tear reservoir that concentrates biomarkers while shielding sensors from corneal contact and eyelid pressure. Sensor color changes are directly observable and quantifiable via a smartphone without specialized readers or user‐specific calibration. Validated using artificial tear fluids, human tear samples, and in vivo models, this platform enables wearable monitoring of DED‐related biomarkers within physiologically relevant ranges, offering a simple and clinically viable approach for tracking ocular surface conditions and associated treatment efficacy.

## Graphical abstract

An electronics‐free smart scleral lens passively monitors dry eye disease‐related tear biomarkers through colorimetric sensing of chloride, pH, and ocular surface temperature. Smartphone‐assisted image analysis enables quantitative interpretation, providing a simple, non‐invasive strategy for ocular health monitoring without integrated electronics.

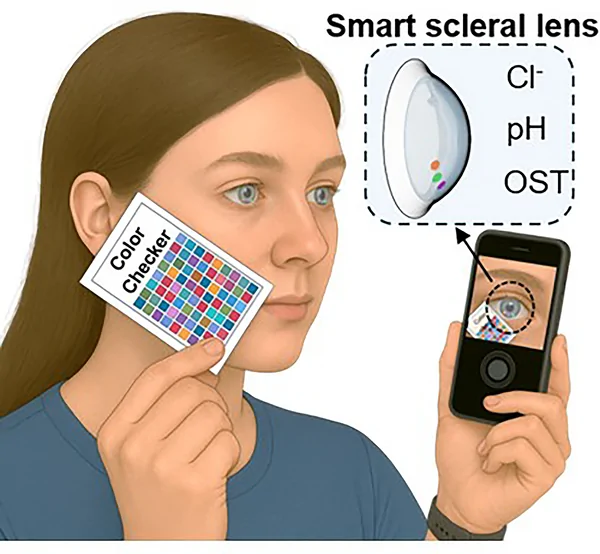

## 1 Introduction

Dry eye disease (DED) is a prevalent and multifactorial ocular surface disorder, affecting over 20 million adults in the United States and more than 344 million individuals globally [1]. It is characterized by tear film instability, hyperosmolarity, inflammation, and neurosensory dysfunction, resulting in ocular discomfort, fluctuating vision, and progressive corneal damage [2, 3, 4, 5, 6, 7]. The episodic and fluctuating nature of DED presents a major diagnostic challenge, as conventional assessments such as the Dry Eye Questionnaire (DEQ‐5), ocular surface staining, and tear film evaluation require clinic‐based procedures and specialized equipment [8, 9]. These assessments offer only point‐in‐time snapshots and often overlook transient physiological changes that occur throughout the day. Frequent clinic visits are often impractical, leading to delayed diagnosis and suboptimal management, particularly for elderly or underserved patients [10]. These limitations highlight the need for non‐invasive, at‐home/real‐world monitoring strategies that empower patients to independently track dynamic ocular changes without relying on clinical supervision [11, 12].

There is growing interest in portable and wearable sensing platforms for non‐invasive, self‐administered monitoring of tear biomarkers in at‐home settings [13, 14, 15, 16, 17, 18]. These systems can track analytes such as glucose, electrolytes (e.g., chloride), and inflammatory proteins (e.g., MMP‐9) [19, 20, 21, 22, 23, 24, 25, 26, 27], and clinical devices like TearLab and I‐PEN provide chairside tear osmolarity measurements for DED diagnosis, though they remain clinic‐centric and rely on trained personnel [28]. Recent work has introduced DED‐focused portable and wearable technologies [29, 30], including microfluidic MMP‐9 tests [31], triboelectric systems for assessing tear‐film instability and blink behavior [32, 33], and ocular wearables using fluorescent chemistries to detect surface inflammation [16, 34, 35, 36, 37]. Despite these advances, their translation to user‐friendly at‐home monitoring remains constrained [38]. Many platforms still require integrated electronics, external readers, or wired components that complicate miniaturization and continuous wear [39, 40], while others depend on manual tear sampling, are susceptible to evaporation or clogging, or use rigid substrates that affect comfort [41, 42]. As a result, there are currently no commercially available devices capable of simultaneously assessing tear Cl^−^, pH, and ocular surface temperature in situ under the same conditions as a wearable ocular platform. Optical sensors offer a simple electronics‐free sensing strategy that enables direct visual readout without electronics [43], yet integrating them into commercial soft contact lenses is difficult due to mechanical mismatch, limited surface area, and the cornea‐conforming geometry that prevents stable tear accumulation essential for reliable sensing [42, 44].

Scleral lenses provide a well‐suited platform to address these limitations. By resting on the sclera and vaulting over the cornea, they create a stable, tear‐filled reservoir between the posterior lens surface and the ocular surface [45, 46]. This vaulted chamber continuously exchanges fresh tear fluid through capillary flow and blinking dynamics, sustaining a biomarker‐rich microenvironment directly above the epithelium that is far more stable and concentrated than the exposed tear film [47]. Importantly, this tear reservoir also creates a protected environment that enables passive optical sensing approaches without requiring integrated electronics or external tear sampling. Although conventional soft contact lenses may be poorly tolerated by some patients with unstable tear films, scleral lenses are clinically used in selected patients with moderate‐to‐severe DED and ocular surface disease to support ocular surface hydration and epithelial protection [48, 49]. Yet despite these advantages, no existing platform has leveraged the intrinsic tear reservoir of a scleral lens for passive, at‐home biomarker monitoring.

Leveraging the unique anatomical advantages of scleral lenses, we present the first electronics‐free smart scleral lens platform that passively incorporates fully self‐contained, multi‐analyte optical sensors directly into the tear reservoir between the lens and cornea. Rather than targeting high‐precision electronic diagnostics, this platform is designed to enable simple monitoring of relative biomarker changes and physiological ranges through intuitive visual readouts. Our design exploits the naturally protected, biomarker‐rich microenvironment created by the scleral lens vault—an area continuously replenished with fresh tear fluid and inherently isolated from eyelid pressure and external contamination. Built upon a commercially available scleral lens (Zenlens, Bausch + Lomb Specialty Vision Products) used in clinical practice for ocular surface management, the platform preserves the established lens form factor while enabling non‐contact, electronics‐free sensing within the tear reservoir. The sensing elements are embedded entirely within the tear reservoir and remain suspended above the epithelium without direct tissue contact, allowing continuous exposure to physiologically relevant tear biomarkers. This enables wearable monitoring of tear Cl^−^, pH, and ocular surface temperature (OST), which are associated with DED‐related changes in tear osmolarity, ocular surface microenvironment, and tear film stability. Their colorimetric responses are visible to the naked eye and can be quantitatively analyzed via smartphone imaging using a customized color reference chart and a machine learning‐based regression model. Validation with artificial tear fluids, analysis of human tear samples from DED subjects, and in vivo feasibility studies in rabbit and canine models collectively demonstrate the potential of this platform as a user‐friendly and clinically compatible approach for at‐home tracking of DED‐related tear biomarker profiles.

## 2 Results

### 2.1 Device Architecture and Biomarker Quantification

Figure 1 provides a schematic overview of the smart scleral lens (SSL) system, designed for non‐invasive, wearable monitoring of ocular health in daily, at‐home settings. The integrated optical sensors provide two complementary at‐home readout modes: (i) qualitative visual assessment using a handheld mirror (Figure 1A), allowing users to inspect color changes directly on‐eye, and (ii) quantitative analysis using smartphone imaging in combination with a compact color reference chart, which standardizes the captured images and enables machine‐learning‐based biomarker estimation (Figure 1C).

**FIGURE 1:**
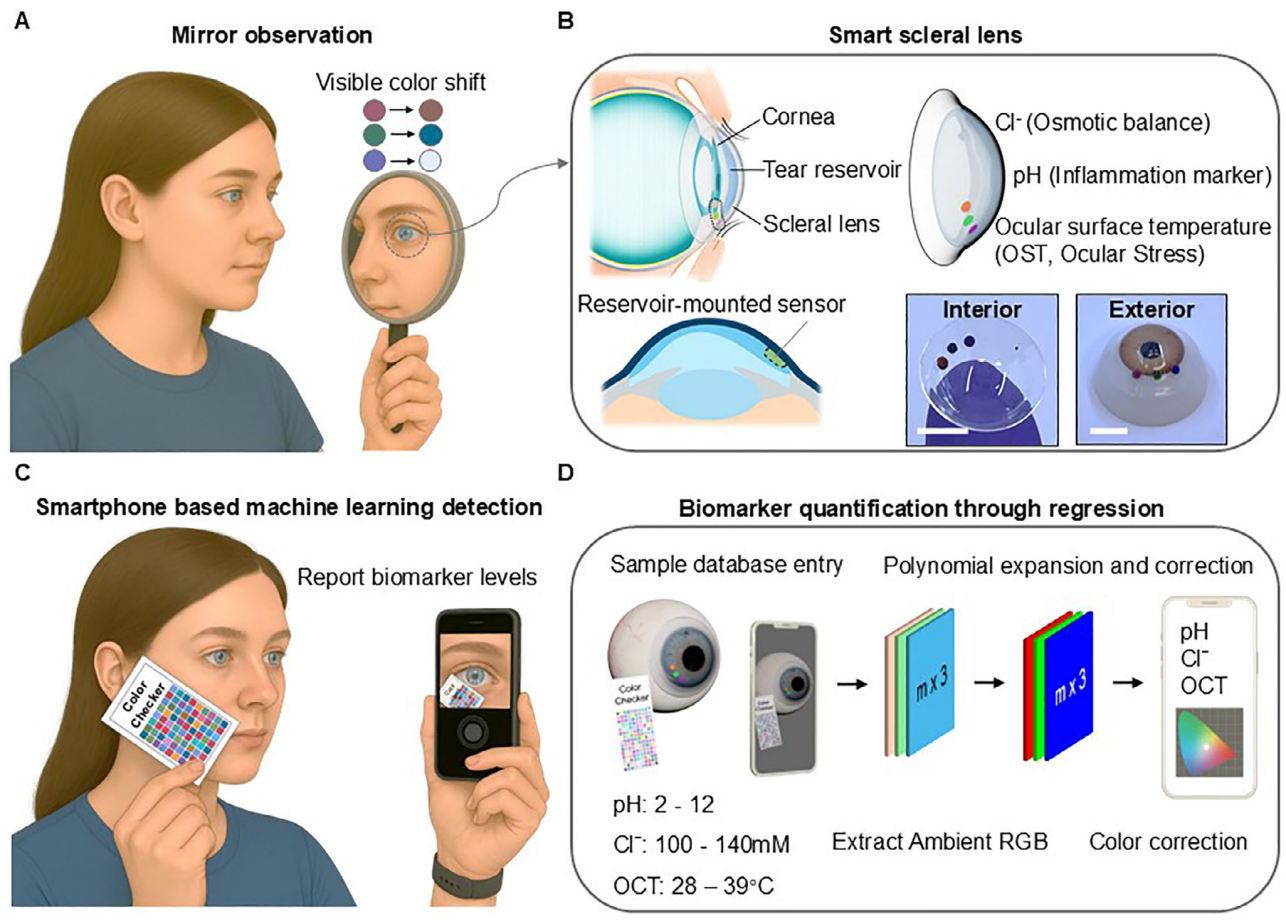
System overview of the smart scleral lens and at‐home readout workflow. (A) Qualitative mirror‐based inspection showing visible sensor color changes. (B) Smart scleral lens architecture with colorimetric sensors for Cl^−^, pH, and OST embedded on the inner surface within the tear reservoir; cross‐section highlights corneal clearance. Interior and exterior device images (scale bars: 5 mm for interior; 1 cm for exterior) demonstrate the conformal lens geometry. (C) Smartphone‐based color‐capture workflow using a printed color reference chart for illumination‐ and device‐independent calibration. (D) Quantification pipeline in which color‐correction models transform raw RGB values into CIE XYZ space and subsequently estimate tear biomarkers (Cl^−^, pH, OST) with cross‐phone, cross‐lighting, and cross‐format robustness.

Figure 1B illustrates the SSL architecture, with optical sensors targeting Cl^−^, pH, and OST strategically embedded along the inner surface of the lens within the tear reservoir. The sensors are thin and suspended above the cornea, ensuring continuous access to fresh tears while avoiding direct corneal contact. This configuration supports on‐eye monitoring of biomarker‐induced color changes in response to physiological fluctuations, with all sensing components completing color development within approximately 15 min under the tested conditions. Insets show interior and exterior lens views, highlighting sensor placement, geometry, and conformity to ocular anatomy while maintaining corneal clearance and wearer comfort.

The selected biomarkers were chosen to reflect critical physiological and pathological features of the ocular surface associated with DED. Cl^−^ concentration serves as the primary biomarker, as it directly reflects tear film osmolarity—a central pathogenic factor in DED that drives hyperosmolar stress, promotes inflammatory cascades, and leads to epithelial cell damage [50]. To complement this, tear pH and OST were incorporated as secondary indicators to provide additional insight into ocular surface health. Tear pH is a sensitive marker of inflammation; in DED, especially in chronic or progressive forms, alkalization of tear fluid is associated with tear film instability and epithelial barrier disruption [51, 52]. OST functions as an indirect but informative metric of evaporative dynamics and ocular surface inflammation, particularly relevant in meibomian gland dysfunction and lipid layer instability [53]. Together, these biomarkers offer a multifaceted assessment of DED pathophysiology. Table S1 summarizes their clinical relevance and reported reference ranges in both healthy individuals and DED patients.

Figure 1D illustrates the color correction model used for biomarker quantification, which transforms setting‐dependent RGB values captured under varying imaging conditions into setting‐independent CIE XYZ values through color correction. To ensure high color accuracy, a multivariate regression framework of color correction was developed using a custom‐designed color reference chart containing biomarker‐specific reference color patches. An overdetermined system was formed from the RGB values extracted from a photograph and their corresponding ground‐truth CIE XYZ values for the reference color chart, and was solved using the Moore‐Penrose pseudoinverse to obtain the transformation matrix converting the acquired RGB values to true CIE XYZ values. The resulting transformation matrix enabled accurate extraction of setting‐independent true colors from lens‐mounted sensors, which were used to quantify Cl^−^, pH, and OST, providing a robust basis for real‐time image‐based biomarker monitoring under typical at‐home lighting.

### 2.2 Cl^−^ Sensor Design, Calibration, and Validation

Optical sensors were integrated into the scleral lens chamber to detect tear Cl^−^, pH, and OST within physiologically relevant ranges. For the fabrication of the Cl^−^ sensor, ion exchange resins were employed to encapsulate the sensing reagents while permitting diffusion of small ions from the surrounding tear fluid. The anion‐exchange resin contains quaternary ammonium functional groups that electrostatically retain the sensing reagents on the porous resin surface, allowing the colorimetric reaction to remain localized while enabling ion diffusion from the surrounding tear fluid. These functionalized resins were then embedded within a poly(2‐hydroxyethyl methacrylate)‐polyethylene glycol (pHEMA: PEG) hydrogel matrix to enhance biocompatibility and ensure robust adhesion to the lens surface, as illustrated in Figure S1. Encapsulation of the sensing beads within the hydrogel matrix prevents dispersion of the resin particles into the tear reservoir while providing a soft and biocompatible interface with the ocular surface. The hydrogel layer remains permeable to water and small ions, allowing efficient analyte transport while mechanically stabilizing the sensing elements within the lens structure.

Cl^−^ is a key electrolyte in tear fluid, typically ranging from 122.86 ± 7.12 mm in healthy individuals [50]. Its dysregulation leads to tear hyperosmolarity, a hallmark of aqueous‐deficient DED, and is closely linked to ocular surface stress and tear film instability, underscoring its value as a diagnostic biomarker [54]. To capture both physiological and pathological fluctuations, we developed a Cl^−^ sensor using silver chloranilate as the responsive chromophore, calibrated over an extended range of 50 to 200 mm to ensure accurate and reliable detection.

To optimize the sensor matrix, silver chloranilate was tested at concentrations ranging from 0 to 0.5 mm. A concentration of 0.5 mm yielded approximately 40% transmittance, providing a clear visual response while maintaining an adequate dynamic range in the visible spectrum (Figure 2B). Figure 2A shows the sensor's color transition from purple to brown as Cl^−^ concentration increases from 50 to 200 mm. This transition arises from the formation of silver chloride (AgCl), which attenuates the silver chloranilate complex and disrupts charge‐transfer interactions. The sensing element operates as a time‐evolving colorimetric indicator that progressively reflects variations in chloride concentration during exposure to tear fluid, with the color response stabilizing within approximately 15 min under the tested conditions. Consequently, the transmittance at 540 nm decreases (Figure 2C), accompanied by increased optical scattering from AgCl precipitates, producing a distinct, concentration‐dependent visual signal. Although other halides can theoretically interact with silver‐based reagents, chloride is the dominant anionic species in tear fluid, while common tear metabolites do not participate in the silver chloranilate reaction [50, 54, 55, 56, 57, 58, 59, 60]. The concentrations of major tear components and their potential interactions with the sensing chemistry are summarized in Table S2.

**FIGURE 2:**
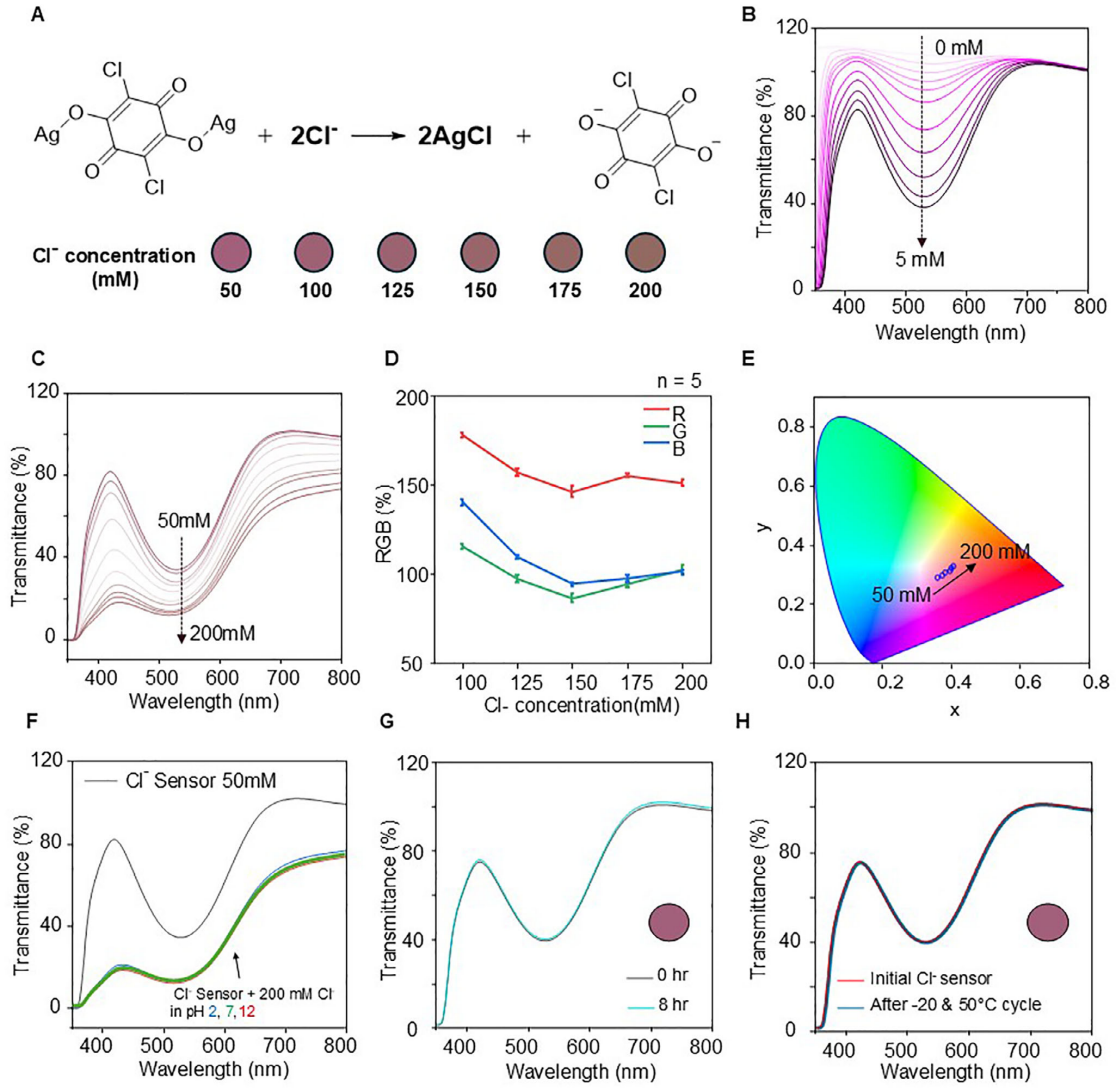
Chloride (Cl^−^) sensor calibration and validation. (A) Visual transition of the sensor from purple to brown as Cl^−^ increases from 50–200 mm. (B) UV–Vis spectra of the silver–chloranilate colorimetric reagent at varying concentrations. (C) Corresponding spectra showing decreased transmittance at 540 nm with increasing Cl^−^. (D) RGB channel responses across the Cl^−^ range (n = 5, mean ± s.d.). (E) CIE xy chromaticity mapping of sensor colors versus concentration. (F) Stability under various pH (pH 2, 7, 12). (G) Spectral stability of the Cl^−^ sensor after 8 h of immersion. (H) Thermal robustness after cycling between −20°C and 50°C.

To calibrate the sensor, chloride‐responsive resin beads were imaged across the full Cl^−^ concentration range. RGB signal variations are shown in Figure 2D, with corresponding CIE xy chromaticity values mapped in the CIE XYZ color space in Figure 2E. Cross‐reactivity with pH was minimal, as sensor output remained stable at pH 2, 7, and 12 (Figure 2F), confirming Cl^−^ specificity. Spectral stability was evaluated over an 8 h period, which is within a typical daily scleral lens wear duration (Figure 2G). The Cl^−^ sensor maintained stable absorbance profiles without abrupt signal degradation after color development. RGB analysis at 0, 2, 4, 6, 8, and 12 h further showed only minor variations, with no abrupt color drift under fixed testing conditions, supporting stable optical readout over the monitored duration (Figure S2). Thermal cycling between −20°C and 50°C further confirmed the optical robustness of the sensing element under temperature variations (Figure 2H).

### 2.3 pH and OST Sensor Design, Calibration, and Validation

The pH sensor was fabricated using the same ion exchange resin and pHEMA: PEG hydrogel strategy used for the Cl^−^ sensor to ensure biocompatibility and strong adhesion within the lens chamber. Following Cl^−^ sensor validation, the pH sensor was evaluated across clinically relevant ranges. Tear fluid in healthy individuals typically exhibits a pH of 6.5–7.6, with near‐neutral pH essential for maintaining ocular surface homeostasis and minimizing epithelial irritation [51]. Deviations from this range are associated with several ocular conditions. Elevated tear pH has been reported in patients experiencing contact lens discomfort and after refractive surgery due to impaired buffering capacity [52, 61], while tear acidification is characteristic of microbial keratitis, where inflammation and bacterial metabolism lower pH [62]. To support broad diagnostic utility, the pH sensor was engineered to detect both acidic and alkaline deviations with high fidelity.

Bromothymol blue (BTB) was selected as the pH‐responsive chromophore due to its distinct and reversible color transition within the physiological pH range. Transmittance testing across BTB concentrations (10–60 µm) identified 50 µm as optimal, yielding ∼20% baseline transmittance without oversaturation (Figure S3). As shown in Figure 3A, BTB transitions from yellow to green to blue as pH increases from 2 to 6 to 12, driven by deprotonation of the phenolic group and corresponding shifts in π‐conjugation and absorbance. Figure 3B confirms this pH‐dependent spectral transition, with the protonated form exhibiting a characteristic absorption band around 427 nm and the deprotonated form showing a characteristic absorption band around 617 nm, corresponding to the observed transmittance changes. The pH‐dependent color response stabilized within approximately 5 min under the tested conditions, enabling rapid optical readout of tear pH changes.

**FIGURE 3:**
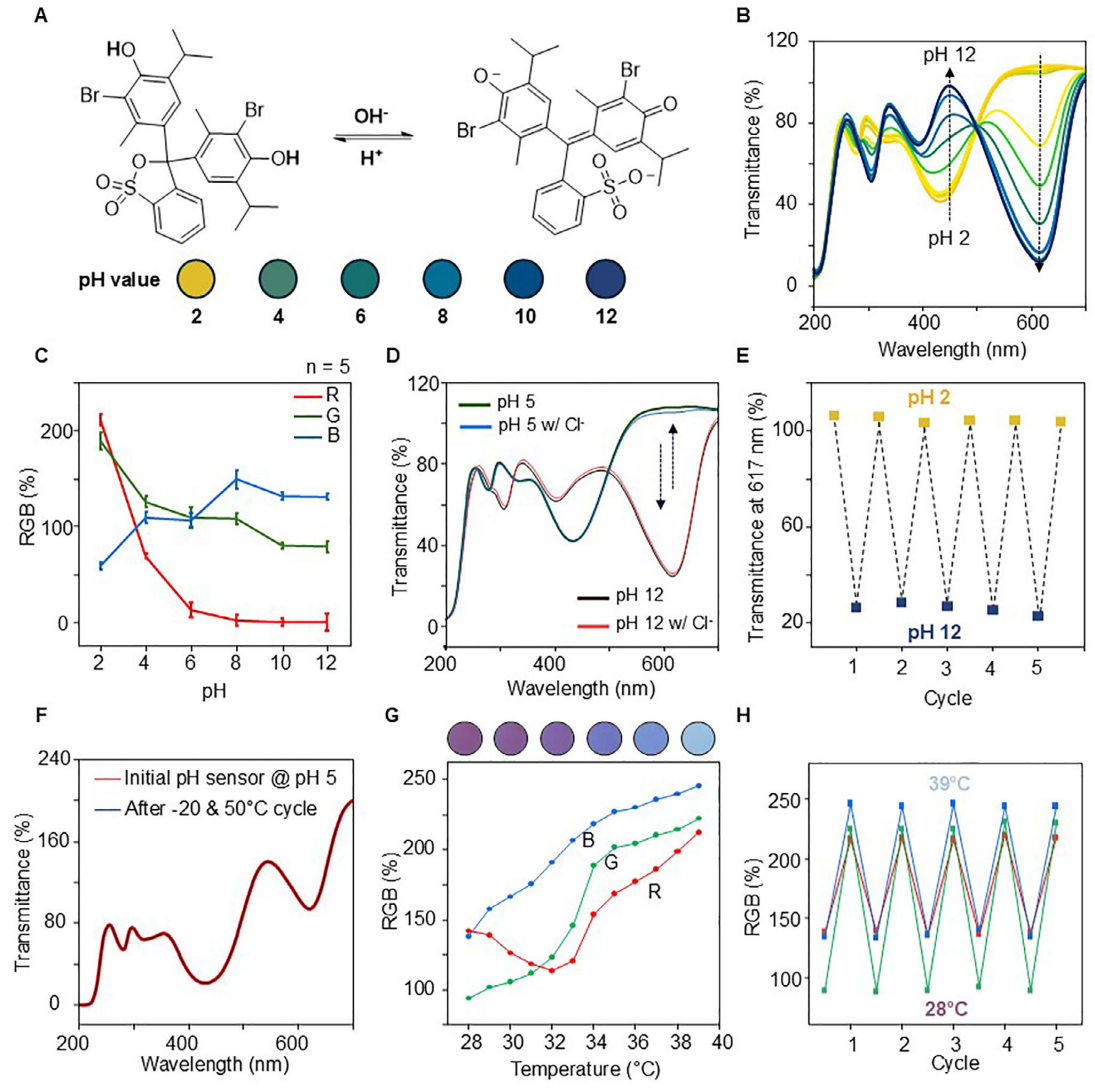
Calibration and validation of the pH and OST sensors. (A) pH sensor response based on the acid‐base interconversion of bromothymol blue (BTB), showing characteristic color transitions across pH 2–12. (B) UV–Vis transmittance spectra of the pH sensor showing pH‐dependent spectral changes, with characteristic absorption features of the protonated and deprotonated BTB forms at approximately 427 and 617 nm, respectively. (C) RGB channel responses of the pH sensor as a function of pH (n = 5, mean ± s.d.). (D) pH‐sensor specificity evaluated by comparing spectra at pH 5 and pH 12 in the presence and absence of 200 mm Cl^−^, confirming minimal ion‐interference effects. (E) Reversibility of the pH sensor during repeated cycling between pH 2 and pH 12, showing stable absorbance at 617 nm. (F) Thermal robustness of the pH sensor after repeated temperature cycling between −20°C and 50°C. (G) RGB channel responses of the OST sensor as a function of temperature. (H) Reversibility of the OST sensor during repeated cycling between 28°C and 39°C, demonstrating stable and repeatable thermal readout performance.

For calibration, BTB‐loaded ion exchange resin beads were imaged across the full pH range. RGB intensity changes are shown in Figure 3C, with CIE xy chromaticity values plotted in the CIE XYZ color space (Figure S4). Figure 3D further validates sensor specificity, showing negligible influence from physiological Cl^−^ levels. Figure S5 evaluates dye retention after immersion by comparing the spectra at pH 5 before and after 8 h, showing no apparent spectral shift or signal loss. Reversibility and optical stability were demonstrated by repeated pH cycling between pH 2 and 12, with minimal change in transmittance at 617 nm (Figure 3E). Thermal robustness was confirmed under repeated cycling between −20°C and 50°C, where the sensor maintained consistent optical properties (Figure 3F).

OST serves as a valuable indicator of tear film stability and evaporative dynamics and is frequently altered in DED. In evaporative DED subtypes, rapid interblink cooling of up to 0.78°C over 10 s has been linked to meibomian gland dysfunction and lipid layer instability [53]. To capture these physiological fluctuations, a thermochromic composite sensor was integrated into the scleral lens chamber. This sensor contained three calibrated thermochromic powders embedded within a polydimethylsiloxane (PDMS) matrix and was affixed to the inner lens surface via in situ polymerization of a polydopamine (PDA) adhesive layer. The sensor exhibited a continuous and visually discernible transition from purple to pale blue across the physiological OST range of 28°C to 39°C. Following temperature variation, the OST sensor reached a stable color response within approximately 1 min under the tested conditions.

Quantitative RGB analysis confirmed a strong temperature‐dependent trend, with blue and green channel intensities increasing consistently with rising temperature (Figure 3G). Corresponding CIE xy chromaticity shifts in the CIE XYZ color space (Figure S6) demonstrated robust and quantifiable optical changes across the physiological temperature range. The OST sensor showed excellent reversibility under repeated cycling between 28°C and 39°C (Figure 3H) and maintained stable optical output in the presence of varying pH and Cl^−^ concentrations (Figure S7). These results confirm both its thermal sensitivity and environmental stability, supporting its suitability for integration into a multifunctional optical scleral lens platform for DED monitoring.

### 2.4 Optimization and Characterization of Hydrogel Matrix for Sensor Integration

To ensure long‐term biocompatibility, signal stability, and strong adhesion to the scleral lens, a pHEMA: PEG hydrogel matrix was developed to encapsulate the Cl^−^ and pH sensing beads. HEMA, a clinically established contact lens material, offers hydrophilicity and oxygen permeability, while PEG400 was added as a plasticizer to enhance softness, stretchability, and wetting behavior—thereby improving adhesion and mechanical compliance [63]. Hydrogel matrices with varying HEMA: PEG400 molar ratios (9:1 to 5:5) were synthesized and evaluated for mechanical and optical properties. All formulations exhibited significantly lower stiffness than commercial scleral lenses (Zenlens; E = 1.16 GPa) and soft contact lenses (ACUVUE OASYS; E = 9.02 ± 0.218 MPa). As PEG400 content increased, Young's modulus decreased, with the softest matrix (5:5) reaching E = 0.14 ± 0.733 MPa (Figure 4A). Transmittance spectra confirmed near‐100% transparency across the visible range for all hydrogel formulations (Figure 4B), indicating minimal impact on optical sensing performance. SEM analysis revealed distinct surface morphologies: the plain hydrogel exhibited a smooth, wrinkled texture indicative of a uniform polymer network, whereas the bead‐loaded hydrogel showed a rough, particulate surface due to embedded sensing elements (Figure S8). These features confirmed successful sensor integration within the hydrogel matrix.

**FIGURE 4:**
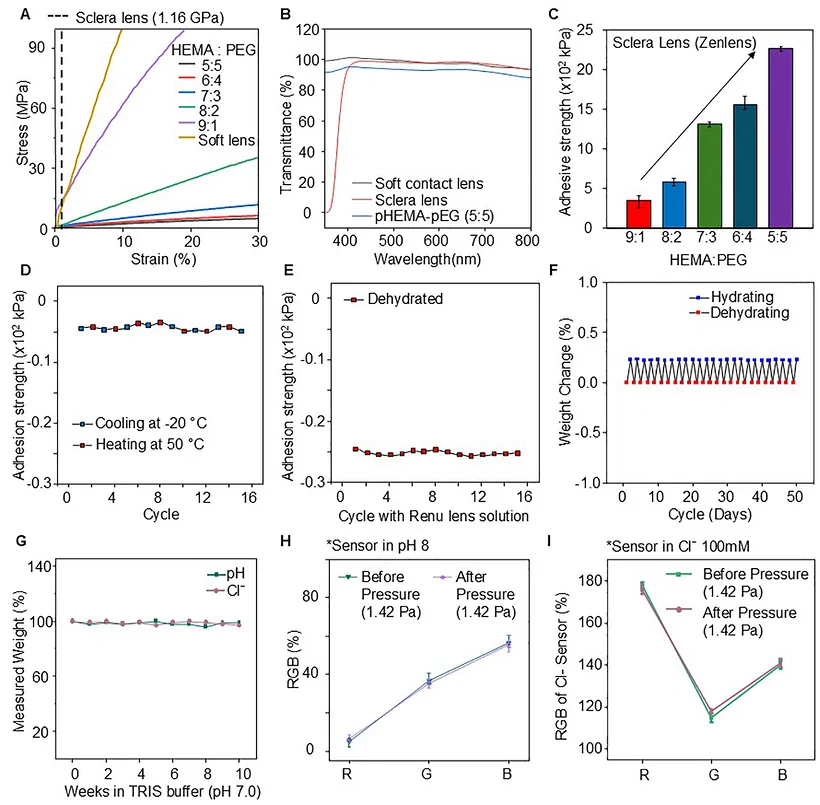
Optimization and characterization of the pHEMA: PEG hydrogel matrix for sensor integration. (A) Mechanical compliance of hydrogels with varying HEMA: PEG400 ratios (9:1 to 5:5), showing decreasing Young's modulus with increasing PEG content and substantially softer matrices than commercial scleral and soft contact lenses. (B) Transmittance spectra demonstrating ∼100% transparency across the visible range for all formulations. (C) Improved adhesion strength to the Zenlens (PMMA) surface improves with PEG400 content. (D) Adhesion retained after 15 thermal cycles between −20°C and 50°C. (E) Adhesion retained after wet‐dry cycling in commercial lens solution. (F) Minimal mass/weight variation over 25 hydration‐dehydration cycles across 50 days. (G) Mass change during 10 weeks of immersion in TRIS buffer (pH 7.0). High RGB signal stability of embedded pH (H) and Cl^−^ (I) sensors under 15 cycles of mechanical compression (1.42 Pa), confirming robustness for extended wear and storage (n = 5, mean ± s.d.).

Adhesion tests demonstrated that increasing PEG400 content improved bonding strength to the Zenlens surface (Figure 4C). This adhesion remained robust after 15 thermal cycles between −20°C and 50°C (Figure 4D), wet–dry cycling in commercial lens solution (Figure 4E), and storage in PBS (Figure S9). The hydrogel also exhibited minimal weight variation over 25 hydration–dehydration cycles spanning 50 days (Figure 4F), supporting strong resistance to cyclic swelling stress. This mechanical durability is attributed to the synergistic effects of PEG400, which enhances interfacial conformal contact and hydrogen‐bonding interactions, and the pHEMA network, which strengthens secondary intermolecular interactions with the lens substrate. Additional thermal annealing (70°C, 8 h) further reinforced adhesion via crosslinking and polymer interpenetration. The stability of the encapsulated chromophores was assessed under mechanical and environmental stress. RGB signal outputs of both pH and Cl^−^ sensors remained consistent during repeated hydration‐dehydration cycles and thermal fluctuations (Figure S10). This stable optical response indicates effective confinement of the sensing reagents within the resin‐hydrogel matrix without detectable signal degradation associated with reagent loss. Long‐term immersion in TRIS buffer (pH 7.0) showed minimal weight loss over 10 weeks (Figure 4G), indicating excellent chemical durability. Moreover, Figure 4H,I demonstrate high RGB signal stability in both sensors under 15 cycles of mechanical compression (1.42 Pa), validating their robustness for extended wear and storage.

### 2.5 In Vivo Assessment of Sensor Fit, Biocompatibility, and Physiological Readout Accuracy

To evaluate the biocompatibility, fit, and physiological functionality of the smart scleral lens sensor, in vivo studies were conducted in purpose‐bred rabbits and dogs. In rabbits, the device was first assessed for wearability. The smart scleral lens remained stably positioned for 1 h without visible irritation relative to a control eye fitted with a bare lens (Figure 5A). Anterior segment OCT imaging (Figure 5B) clearly shows the distinct textures of the hydrogel‐based Cl^−^ and pH sensors and the PDMS‐based OST sensor. The sensor disks have a diameter of approximately 2.5 mm, with hydrogel‐based Cl^−^ and pH sensors exhibiting a thickness of ∼250–300 µm after hydration and the PDMS temperature sensor having a thickness of ∼250 µm. Sensors were positioned in the peripheral region of the scleral lens, outside the central optical zone, following our previous smart contact lens design strategy to minimize visual interference during wear [16]. Each sensor maintains a visible gap above the corneal surface, confirming that the sensors remain suspended within the tear reservoir and vault the corneal epithelium without direct pressure, an essential requirement for long‐term ocular safety. Biocompatibility of the sensing components was further assessed in rabbits (n = 6) over 24 h by integrating identical sensor elements into a soft contact lens, as the available scleral lens geometry did not match rabbit eyes. This approach preserved the sensing materials while avoiding fit‐related irritation. To improve retention, a partial temporary tarsorrhaphy was performed. AS‐OCT imaging after 1 h (Figure S11A) showed mild deformation of the soft lens but normal corneal apposition. Clinical slit‐lamp and fluorescein staining images (Figure S11B) revealed no signs of epithelial injury after 24 h. Histopathology (Figure 5C) demonstrated minimal to mild, comparable lesions in both sensor‐integrated and control lenses, primarily mild epithelial hyperplasia and occasional lymphoid follicles with inflammation scores summarized in Table S3. No evidence of epithelial damage or abnormal inflammatory response attributable to the sensing materials was observed. These findings indicate good ocular tolerance and biocompatibility of the integrated sensing components under the tested conditions.

**FIGURE 5:**
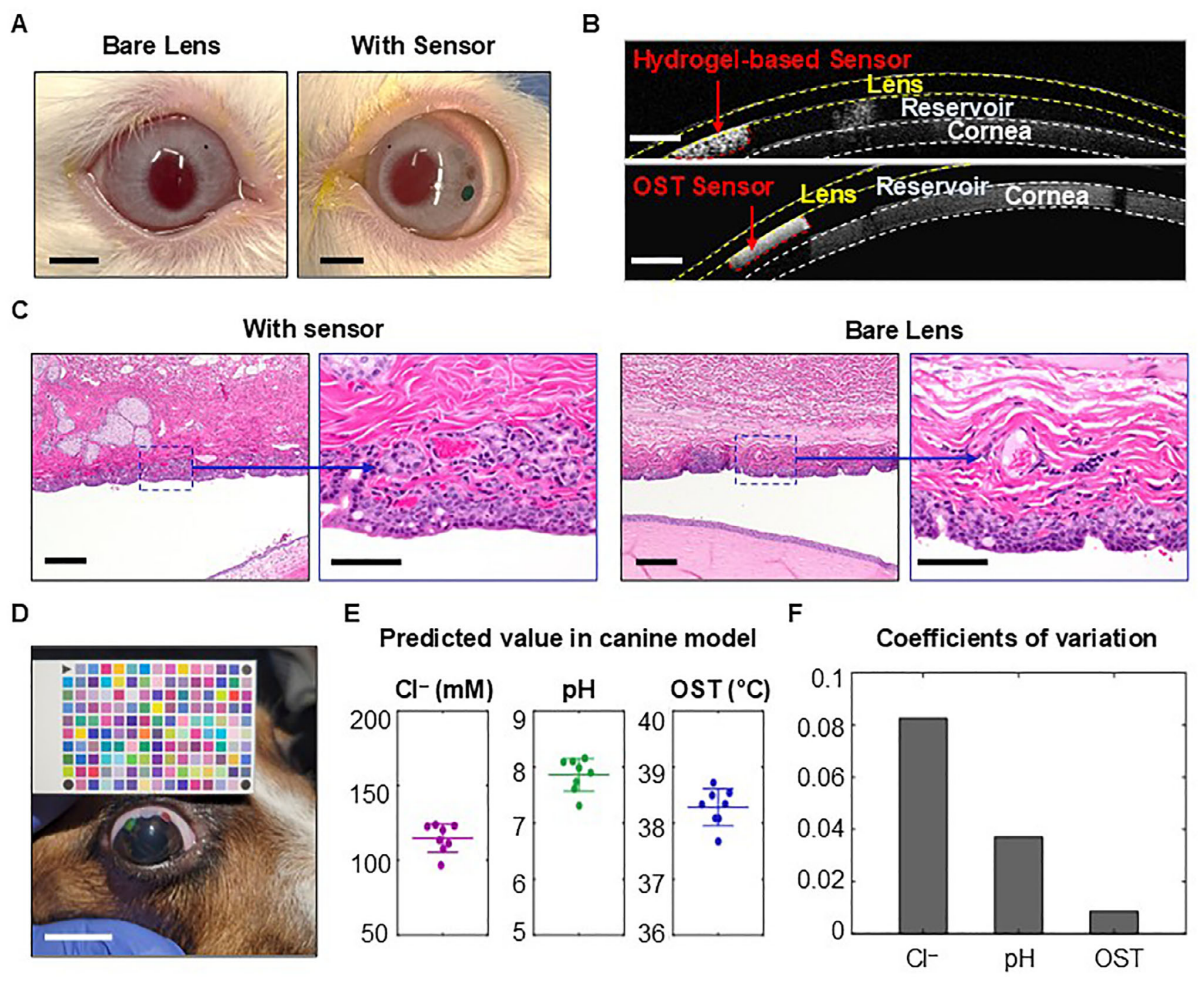
In vivo assessment of sensor fit, biocompatibility, and physiological readout accuracy. (A) Rabbit wear test comparing a bare scleral lens and a sensor‐integrated scleral lens, both showing stable centration during blinking and eye movement (scale bar: 5 mm). (B) AS‐OCT cross‐sections confirming proper corneal vaulting of the SSCL, with a distinct reservoir gap between the hydrogel‐based sensor or OST sensor and the corneal surface, indicating a non‐contact fit (scale bar: 1 mm). (C) Representative H&E‐stained histological sections of rabbit cornea after 24 h of wear showing mild epithelial erosion and minimal mixed inflammatory infiltration with localized edema at the limbus for both SSCL and bare lens groups (scale bars: overview images 200 µm; magnified regions 50 µm). (D) A canine eye fitted with the smart scleral lens and printed color reference chart for smartphone‐based image capture and quantitative color analysis (scale bar: 2 cm). (E) Predicted tear‐film chloride concentration, pH, and ocular surface temperature (OST) from eight captured images using the on‐lens color‐reference calibration scheme (n = 8; mean ± s.d.). (F) Coefficients of variation (< 0.1) for all analytes (n = 8), demonstrating robust, quantitative measurements independent of smartphone device and illumination conditions.

To assess physiological readout accuracy in a model with human‐like ocular dimensions, the smart scleral lens was next evaluated in vivo in a 15‐month‐old female beagle. The lens was worn under ambulatory conditions (Movie S1) and imaged alongside a custom‐designed color reference chart to enable quantitative analysis (Figure 5D). A machine‐learning‐based regression model extracted RGB values using a calibrated reference chart (design shown in Figure S12), reflecting the intended at‐home workflow in which users capture a smartphone image for automated readout. This chart‐based color correction strategy builds on our previously validated framework for recovering setting‐independent CIE XYZ values across different smartphone models, illumination conditions, and image file formats [64, 65]. In the present study, the same principle was adapted into a lens‐compatible color reference chart to support consistent and quantitative readout under the intended smartphone imaging workflow. Color responses of the Cl^−^, pH, and OST sensors were mapped into distinct CIE xy chromaticity gamuts (Figure S12A), supplemented by 12 off‐gamut primary colors (Figure S12B) and consolidated into a 146‐color reference set (Figure S12C). The CIE XYZ values of the defined reference colors within each sensor gamut were converted to sRGB for the printable final chart layout (Figure S12D), which was then printed using an ICC‐calibrated workflow to produce the final reference chart. Using the gamut‐mapping strategy in Figure S12, in vivo sensor colors yielded quantitative outputs: Cl^−^ = 113.48 mm, pH = 7.73, and OST = 38.08°C—all within normal physiological ranges for dogs.^49–51^ The corresponding statistical analysis (Figure 5F) shows coefficients of variation < 0.1 across all modalities, demonstrating strong consistency and highlighting the importance of color correction for lighting‐, device‐, and file format‐independent quantification. These results demonstrate the feasibility of the electronics‐free sensing platform for capturing physiologically relevant tear biomarker variations during on‐eye operation. Building on this in vivo feasibility assessment, we next evaluated the quantitative accuracy and imaging‐condition robustness of the same color correction‐based workflow using artificial tear fluids with predefined analyte values and controlled‐temperature conditions.

### 2.6 Quantitative Validation of Smart Scleral Lenses Using Artificial and Human Tear Samples

Prior to human tear sample analysis, the quantification algorithm for each sensor was validated using artificial tear fluids (ATF) with defined analyte values and tear‐relevant background components. Two artificial tear formulations, ATF1 and ATF2, with defined pH/Cl^−^ values of 7.0/110 and 8.0/150 mm, respectively, were used as reference samples for validating Cl^−^ and pH quantification, while OST sensing was validated under controlled temperature conditions. Across different imaging settings, the sensor‐derived values showed good agreement with the reference values, with root mean square errors (RMSEs) of 4.42 mm, 0.21 pH units, and 0.92°C for Cl^−^, pH, and OST, respectively, and coefficients of variation not exceeding 0.04 (Figures S13–S15). Sensor selectivity was further evaluated against tear‐relevant interferents included in the ATF matrix, including Na^+^, K^+^, Ca^2^^+^, lactate, glucose, urea, and bovine serum albumin (Figure S16). The Cl^−^ sensor showed a distinct response in Cl^−^‐containing samples, including ATF1 and ATF2, while non‐chloride interferents induced minimal signal variation. For the pH sensor, individual interferent solutions and ATF1 were maintained at pH 7 and showed similar readouts, whereas ATF2 produced the expected response corresponding to pH 8. The OST sensor was tested at room temperature for all conditions and showed negligible variation across the interferent solutions, indicating minimal chemical interference with the thermochromic readout. These results support the analytical selectivity of the sensing platform in tear‐relevant chemical environments.

To assess sensor accuracy and reproducibility, we performed quantitative analysis of human tear samples collected from 10 adults clinically diagnosed with mild to severe DED. DEQ‐5 questionnaire scores (Figure 6A) classified participants as mild (scores ≤ 5), moderate (6–11), or severe (≥ 12) [66]. Representative corneal and conjunctival fluorescein staining images (Figure 6B) demonstrate progressive ocular surface damage from moderate to severe DED, with more extensive punctate epithelial erosions and conjunctival staining observed in participants with higher DEQ‐5 scores. Additional staining images are provided in Figure S17.

**FIGURE 6:**
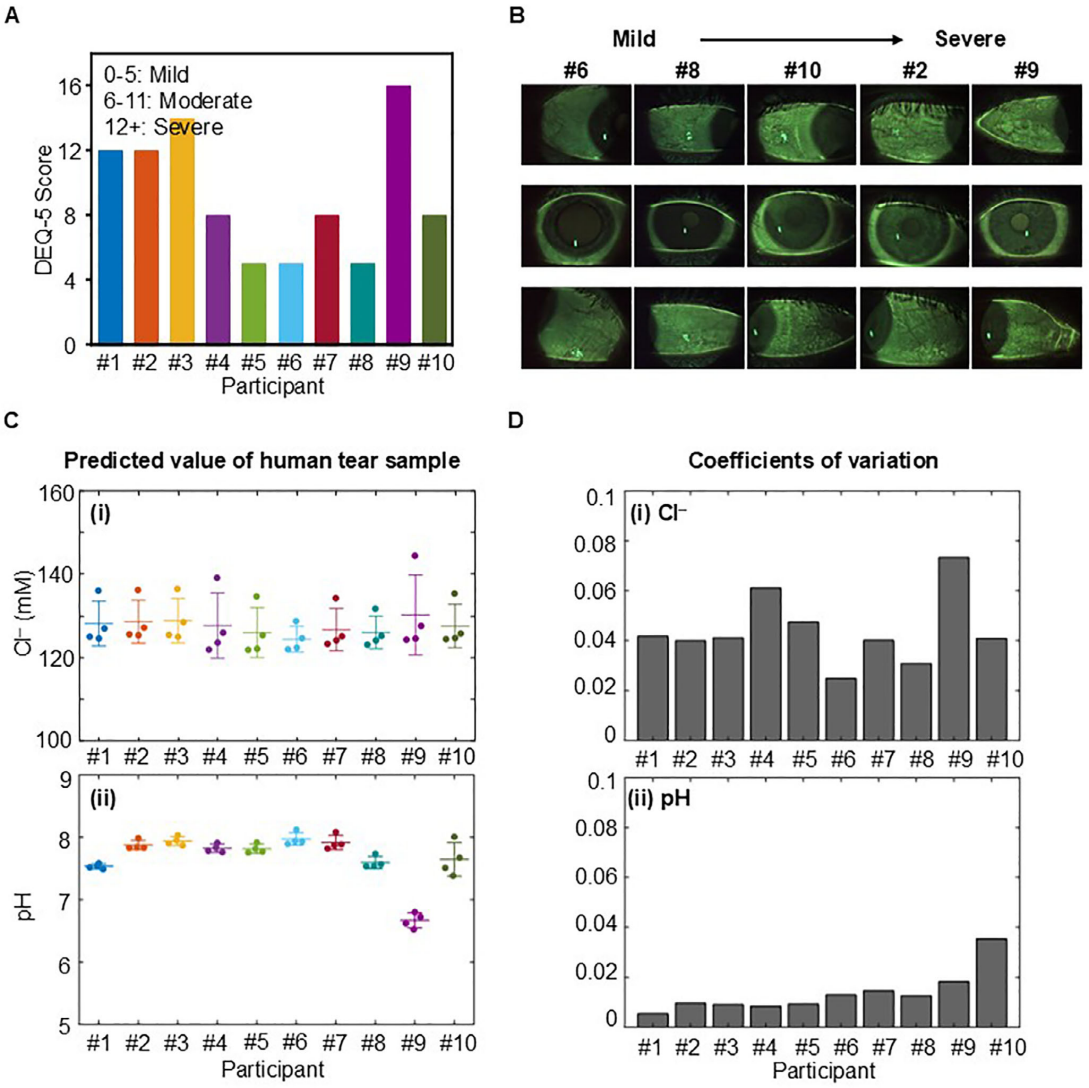
Quantitative analysis of human tear samples with the smart scleral lens. (A) DEQ‐5 scores for ten participants (mild ≤5, moderate 6–11, severe ≥12). (B) Representative corneal and conjunctival fluorescein staining images ordered from mild to severe DED. (C) Image‐based predictions for (i) Cl^−^ concentration and (ii) pH for each subject (n = 4 measurements per subject, mean ± s.d.). (D) Coefficients of variation for (i) Cl^−^ and (ii) pH (n = 4 per subject), showing low inter‐sample variability (Cl^−^ < 0.074; pH < 0.035).

The predicted Cl^−^ concentrations and pH values are summarized in Figure 6C. For Cl^−^ concentration (i), all participants exhibited elevated levels compared to normal physiological ranges, with mean values of approximately 124.3–130.1 mm, consistent with the hyperosmolar tear profile characteristic of DED. Participants with higher DEQ‐5 scores and more severe staining (#3: 130.1 mm; #9: 128.7 mm) exhibited higher Cl^−^ levels compared to those with milder symptoms (#6: 124.3 mm; #8: 125.9 mm). For pH (ii), most participants demonstrated mildly alkaline tear pH values, while subjects #1, #8, #9, and #10 remained within the normal physiological range. This pattern aligns with DED pathophysiology, wherein tear‐film hyperosmolarity elevates Cl^−^ across patients, and evaporative loss often causes mild pH alkalinization; however, intact buffering capacity or aqueous‐deficient DED can limit pH shifts despite electrolyte elevation.

Figure 6D presents the coefficients of variation (CVs) for each subject, with Cl^−^ measurements showing CVs < 0.074 and pH measurements < 0.035. These low CVs indicate high reproducibility in RGB‐to‐concentration mapping and underscore the robustness of the color correction method in minimizing inter‐setting variability, independent of imaging devices, light conditions, and image file formats.

## 3 Discussion

We present an electronics‐free optical sensing platform integrated into commercial scleral lenses for noninvasive, wearable, and at‐home monitoring of DED‐related tear biomarker profiles. The system leverages the stable tear reservoir formed between the scleral lens and cornea to enable wearable assessment of tear Cl^−^, pH, and OST tear biomarkers associated with DED‐related changes in tear osmolarity, ocular surface microenvironment, and tear film stability. Sensors are embedded within a biocompatible hydrogel matrix on the inner lens surface, enabling passive on‐eye detection without affecting comfort or visual clarity. Quantification is achieved through smartphone imaging and machine‐learning‐based RGB analysis, eliminating the need for on‐board electronics, power sources, or laboratory instrumentation. Although chloride, pH, and ocular surface temperature were used here as model biomarkers, the sensing architecture is adaptable and can be configured to detect a broader range of chemical or protein markers relevant to DED and other ocular surface diseases.

Validation using artificial tear fluids, ex vivo tear samples, and in vivo rabbit and canine models supports the analytical performance, ocular compatibility, and on‐eye feasibility of the platform under controlled and physiologically relevant conditions. This platform provides a clinically compatible and user‐friendly approach for longitudinal tracking of DED‐related tear biomarker changes, enabling both qualitative assessment through direct visualization and quantitative readout via smartphone imaging. By supporting objective monitoring of tear biomarker profiles over repeated at‐home measurements, the system may provide information that complements symptom reporting and supports future personalized ocular surface management.

Our approach combines passive optical sensors with smartphone‐based quantification to enable a power‐free and wearable sensing platform suitable for at‐home biomarker monitoring. This simplified architecture reduces system complexity while preserving practical monitoring utility, making it well suited for episodic self‐monitoring and longitudinal trend tracking. Compared to microfluidic and electrochemical sensing platforms, which often require powered circuitry, laboratory processing, or tear collection devices, the proposed platform provides a more accessible and user‐friendly alternative for longitudinal tracking of tear biomarkers. While electronics‐based sensing systems are typically optimized for high‐precision clinical diagnostics, the proposed platform represents an electronics‐free strategy designed for simple and accessible monitoring of physiological changes in everyday settings, serving as a complementary approach for repeated at‐home monitoring and clinician‐guided interpretation.

Despite the advantages afforded by scleral lenses, including a protected and biomarker‐rich tear microenvironment, their use comes with practical considerations. Scleral lenses require individualized fitting, may be less comfortable for certain users, and necessitate proper handling during insertion and removal. These factors underscore the need to balance clinical benefit with user tolerability, and future clinical studies will be needed to evaluate patient selection criteria, wear comfort, and practical use conditions for sensor‐integrated scleral lenses in DED populations. Additionally, the current platform monitors only three biomarkers and does not assess inflammatory cytokines, protein markers, or osmoprotectants that may provide complementary biological and clinical insights. Because the present design is intended for single‐day or episodic use, future studies will be needed to evaluate extended wear performance, long‐term biocompatibility, and sensor durability. Additional clinical studies incorporating healthy controls and disease‐control cohorts will also be important to determine how these biomarker profiles distinguish DED from other ocular surface conditions with potentially overlapping signatures, such as infection, allergy, or conjunctivitis.

Future work will include systematic cytotoxicity and long‐term biocompatibility evaluations following established biomedical device testing standards to further support clinical translation of the platform. Longitudinal clinical validation in human subjects will also be conducted to correlate sensor outputs with patient‐reported symptoms and standard diagnostic metrics, including comparisons with established clinical assays to evaluate quantitative agreement under real‐world conditions. Larger datasets collected across diverse imaging devices and lighting environments will further improve the robustness of the color‐correction model and reduce potential overfitting. Integration with cloud‐based analytics could further enable remote data review, and longitudinal trend analysis, supporting remote DED management within teleophthalmology frameworks. Expanding the sensor library to incorporate multiplexed chemical and protein‐based markers may broaden the platform's monitoring scope beyond DED to encompass a wider range of ocular surface disorders.

## 4 Methods

### 4.1 Materials

All chemicals were of analytical grade and used without further purification. The following reagents were obtained from Sigma–Aldrich: 2‐hydroxyethyl methacrylate (HEMA), benzoyl peroxide (BP), polyethylene glycol (PEG 400), bromothymol blue (BTB), silver chloranilate, sodium chloride (NaCl), TRIS buffer, PBS buffer, and anion exchange resin (Amberlite HPR4811 Cl Anion Exchange Resin). Deionized (DI) water and methanol were also purchased from Sigma‐Aldrich. Polydimethylsiloxane (PDMS, SYLGARD 184) was supplied by Dow Chemical Company. Thermochromic Blue 33, Thermochromic Red 31, and Thermochromic Yellow 37 were purchased from Nano I&C Co. Commercial scleral lenses (Zenlens, Bausch + Lomb Specialty Vision Products) with diameters of 14.8 and 16.0 mm were used as the platform for sensor integration. Commercial soft contact lenses (Acuvue Oasys, Johnson & Johnson, Inc.) with a diameter of 14.3 mm were used as the platform for sensor integration in the 24‐h animal study.

### 4.2 Assembly and Fabrication of Optical Scleral Lens Sensors

To fabricate dye‐loaded ion exchange resin microbeads for Cl^−^ and pH sensing, two dye‐resin systems were prepared using similar protocols. For the chloride sensor, 0.5 mm silver chloranilate was dissolved in DI water, followed by the addition of 100 mg of resin microbeads. After 1 h of stirring, the beads were filtered, washed thoroughly with DI water, and dried at 70 °C for 2 h. For the pH sensor, 10 mm bromothymol blue (BTB) was dissolved in a 7:3 (v/v) mixture of deionized (DI) water and methanol. After complete dissolution, 563 mg of anion exchange resin powder was added and stirred at room temperature for 1 h to promote ionic interactions and dye encapsulation. The resulting microbeads were filtered, washed with methanol to remove unbound dye, and dried at 70°C for 2 h. Chromogenic pHEMA–PEG hydrogels were synthesized to encapsulate the microbeads. A solution of 10 mg benzoyl peroxide (initiator) in 3 mL DI water was prepared, followed by addition of HEMA and PEG400 at specified ratios. For a 5:5 molar ratio formulation, 4.969 g PEG (12.365 mmol) and 1.609 g HEMA (12.365 mmol) were added. The solution was vortexed, sonicated for homogeneity, and mixed with sensing beads. The precursor was cast into molds, degassed, and thermally polymerized at 80°C for 1 h. Circular hydrogel sensors were punched out and affixed to the inner surface of scleral lenses. The resulting sensor disks had a diameter of 2.5 mm and a thickness of approximately 250–300 µm after hydrogel hydration. The lenses were then subjected to an additional thermal curing step at 80°C for 1 h to reinforce bonding. For temperature sensing, blue, yellow, and red thermochromic dyes were mixed at a 3:2:2 mass ratio and blended into PDMS at 2.5 wt.% total dye content (20:1 base to curing agent). The mixture was homogenized using a Thinky mixer for 15 min, spin‐coated onto glass (250 µm thickness), and cured at 80°C for 2 h. Circular sensors were fabricated using biopsy punches (diameter 2.5 mm) and floated onto a 2 mg/mL dopamine hydrochloride solution (10 mm Tris, pH 8.5) to allow in situ polymerization of a polydopamine (PDA) adhesive layer. After ∼12 h, the sensors were annealed onto the lens surface at 60°C for 30 min to complete PDA bonding.

### 4.3 Optical Characterization of Sensors

Sensor performance was evaluated using a Cary 6000i UV–Vis–NIR spectrophotometer to monitor color transitions under varying Cl^−^ concentrations and pH. For calibration, Cl^−^ and pH sensors were each immersed in standard buffer solutions for 30 min before testing. Optical transmittance of the synthesized hydrogels, a commercial soft contact lens (ACUVUE OASYS 1‐Day, Johnson & Johnson Vision), and a commercial scleral contact lens (Zenlens, Bausch & Lomb) was measured across the visible spectrum. Optical responses of Cl^−^, pH, and OST sensors embedded in the lens were quantified by preparing a series of samples across relevant physiological ranges. Specifically, Cl^−^ sensors at concentrations of 25, 50, 100, 110, 135, 150, and 200 Mm, pH sensors were evaluated from pH 4 to 10 at 1 pH unit increments, and OST sensors from 28°C to 40°C at 1 °C intervals. Photographs of each sensor were acquired using both Android (Samsung Galaxy S21) and iOS (Apple iPhone 12 Pro) smartphones under 5600 K white LED illumination (18‐Inch LED Ring Light Kit, NEEWER). To ensure consistent color measurements across device and lighting variability, each sensor was imaged alongside Macbeth ColorChecker. Images were saved in both RAW (DNG) and JPEG formats. Using Macbeth ColorChecker, RGB values extracted from images of the samples were converted into setting robust CIE XYZ and CIE xy chromaticity values to assess possible color variations in the Cl^−^, pH, and OST sensors.

### 4.4 Environmental and Mechanical Stress Testing

To evaluate sensor durability and performance under potential user mishandling scenarios, the adhesion and RGB signal stability of the sensor‐integrated scleral lenses were tested under various mechanical and environmental stress conditions. For each condition, five specimens were tested over 15 cycles, and three RGB measurements were recorded and averaged per cycle. For dehydration‐rehydration testing, specimens were alternately immersed in PBS buffer (pH 7.0) and Renu Advanced Formula lens solution (Bausch + Lomb) for 12 h each, followed by drying in a desiccator containing anhydrous calcium chloride for another 12 h. This cycle was repeated up to 20 times to simulate repeated wet–dry exposure. In temperature cycling tests, specimens were exposed to alternating high (50°C) and low (−20°C) temperatures for 12 h per cycle. After each cycle, RGB values were assessed using photographs taken with an iPhone 12 Pro (Apple, USA). For pressure testing, the sensors were subjected to a compressive load of 1.42 Pa, with optical output measured from smartphone‐captured images. Each condition was tested in triplicate using five independent sensor specimens.

### 4.5 Adhesive Strength Evaluation

The adhesive strength between the pHEMA–PEG hydrogel matrix and the bare scleral contact lens surface was quantified using a custom‐modified mechanical peeling apparatus (ESM303; Mark‐10) equipped with a high‐resolution force gauge (±0.25%). For testing, scleral lenses were cut into rectangular specimens (4 × 10 mm^2^) and bonded to the chromogenic resin sensor‐embedded hydrogel substrate. The assembled construct was affixed onto the horizontal stage of the apparatus using double‐sided Kapton tape, and vertical peeling was conducted at a constant speed of 0.4 mm/s. Force and displacement were continuously recorded throughout the peeling process. Each test was repeated ten times per specimen, and average values were used for subsequent analysis. The peeling strength (τ) was calculated using the following equation:

(1) $$\tau =\frac{F}{L}$$

where F is the measured peeling force and L is the length of the contact interface. To assess the robustness of the adhesive bond under various environmental stressors, specimens were subjected to three treatment conditions: (1) disinfection with a commercial hydrogen peroxide‐based cleansing kit (3% H_2_O_2_, Clear Care; Alcon, Inc.) for 12 h; (2) dehydration in ambient air at 25 °C and 40% relative humidity for 1 h; and (3) exposure to thermal stress via immersion in saline solution maintained at 75 °C for 30 min. Peeling strength measurements were conducted after each treatment to evaluate durability under clinically relevant handling scenarios.

### 4.6 Statistical Learning of Polynomial Color Correction

Color correction algorithms compute true CIE XYZ values, which are independent of device models, light conditions, and image formats (i.e., setting‐independent), from RGB values acquired under an arbitrary setting (i.e., setting‐dependent). To achieve a high level of color accuracy, statistical learning of polynomial color correction was performed using Macbeth ColorChecker or the designed color reference chart. The mathematical relationship between the setting‐independent CIE XYZ under CIE illuminant E and the setting‐dependent acquired RGB can be obtained:

(2) $$i_{3\times 1}=T_{3\times 3}\;d_{3\times 1}$$

where *d*_3 × 1_ = [*r*, *g*, *b*]^T^  is a vector corresponding to the RGB color values acquired from the camera, *i*_3 × 1_ = [*x*, *y*, *z*]^T^  is a vector corresponding to the CIE XYZ values under CIE illuminant E, and *T*_3 × 3_ is a transformation matrix that converts the setting‐dependent acquired RGB values to the setting‐independent CIE XYZ values, respectively. Specifically, the color correction algorithm aims to calculate *T*_3 × 3_. To change the underdetermined problem (Equation 2) into an overdetermined problem that can be stably solved by a least‐squares method, we utilized the ground truth CIE XYZ (under CIE illuminant E) and acquired RGB values from *m* reference color patches in Macbeth ColorChecker or the optical lens chart. As a training dataset, *D*_3 × *m*_ and *I*_3 × *m*_ were constructed by adding *d*_3 × 1_ and *i*_3 × 1_ from *m* reference color patches. The relationship in Equation 2 can be rewritten:

(3) $$I_{3\times m}=T_{3\times 3}\;D_{3\times m}$$

We established a framework of multivariate polynomial regression (polynomial color correction), incorporating polynomial or root‐polynomial expansion terms which were derived from R, G, and B values [67, 68, 69, 70]. Importantly, assuming a nonlinear relationship between the ground truth CIE XYZ and acquired RGB values enabled accurate and precise color correction of CIE XYZ values [67, 68]. To achieve high‐fidelity color accuracy, *D*_3 × *m*_ was expanded to *D*_*e* × *m*_ such that:

(4) $$I_{3\times m}=T_{3\times e}\;D_{e\times m}$$

where *D*_*e* × *m*_ can be expressed:

(5) $$D_{e\times m}={\left[\begin{array}{cccccc} r_{1} & g_{1} & b_{1} & \text{…} & r_{1}^{i}g_{1}^{j}b_{1}^{k} & \sqrt[{i+j+k}]{r_{1}^{i}g_{1}^{j}b_{1}^{k}}\\ \vdots & \vdots & \vdots & \vdots & \vdots & \vdots \\ r_{m} & g_{m} & b_{m} & \text{…} & r_{m}^{i}g_{m}^{j}b_{m}^{k} & \sqrt[{i+j+k}]{r_{m}^{i}g_{m}^{j}b_{m}^{k}} \end{array}\right]}^{T}\;$$

where *i*, *j*, and *k* represent degree of expansion.

We obtained the least‐squares solution for the expanded transformation matrix in Equation 4 by using the Moore‐Penrose pseudoinverse method [71]:

(6) $$T_{3\times e}=I_{3\times m}\;{\left[D_{e\times m}\right]}^{+}$$

where + denotes the pseudoinverse. Finally, the transformation matrix *T*_3 × *e*_ mathematically computes setting‐independent CIE XYZ values from setting‐dependent acquired RGB values.

### 4.7 Color Measurement of Integrated Sensors

Possible color variations of the integrated Cl^−^, pH, and OST sensors were systematically evaluated in the lens platform. For the pH sensor, seven samples were prepared across a range from pH 4 to 10 in 1‐unit intervals. For the OST sensor, thirteen samples were tested at discrete temperatures from 28 °C to 40 °C with 1 °C intervals. For the Cl^−^ sensor, seven samples were prepared with chloride concentrations of 25, 50, 100, 110, 135, 150, and 200 mm. To ensure stable color development across all sensing components, images were acquired 15 min after sensor exposure to the corresponding test conditions. Each sample was photographed alongside Macbeth ColorChecker using both Android (Samsung Galaxy S21) and iOS (Apple iPhone 12 Pro) smartphones. Photos were captured in both RAW (DNG) and JPEG formats under white LED illumination with a color temperature of 5600 K (18‐Inch LED Ring Light Kit, NEEWER). Macbeth ColorChecker enabled reliable preliminary analysis of color variation for each sensor by providing standardized CIE XYZ and CIE xy chromaticity values, thereby reducing variability from devices, light conditions, and image formats.

### 4.8 Gamut Determination – Cl^−^, pH, and OST

We defined three distinct color gamuts highly relevant to color variations of the Cl^−^, pH, and OST sensors within the scleral lens. For the pH sensor, RGB values were extracted from 75 different locations within each sensor area for each of the seven samples, with multiple sampling locations used to accurately capture the spatial distribution of color variations across the sensor surface, obtaining a total dataset of 525 RGB values. For the OST sensor, RGB values were collected from 75 different locations within each sensor area for each of the thirteen samples, resulting in a comprehensive dataset of 975 RGB values. For the Cl^−^ sensor, we extracted RGB values from 75 locations within each sensor area for each of the seven samples, generating a dataset consisting of 525 RGB values. For the RGB datasets of the Cl^−^, pH, and OST sensors, CIE XYZ values were obtained using the transformation matrix for polynomial color correction in Equation 6, calibrated with Macbeth ColorChecker.

To observe the CIE XYZ values independent of their brightness, the CIE xy chromaticity values were calculated [72]. For the pH sensor, we observed that the 525 CIE xy chromaticity values formed a local group in the CIE xy chromaticity diagram. In particular, a distinct and narrow color gamut (i.e., pH gamut) was defined with six primary points of the CIE xy chromaticity: (x,y) = (0.17,0.18), (0.25,0.42), (0.43,0.50), (0.49,0.45), (0.32,0.34), and (0.21,0.16). For the OST sensor, the 975 CIE xy chromaticity values formed a local group, defining a unique color gamut (i.e., temperature gamut) with four primary points of the CIE xy chromaticity: (x,y) = (0.20,0.14), (0.25,0.26), (0.33,0.33), and (0.30,0.19). In the same manner, the 525 CIE xy chromaticity values of the Cl^−^ sensor also exhibited a local cluster such that another distinct color gamut (i.e., chloride gamut) can be defined with four primary points of the CIE xy chromaticity: (x,y) = (0.32,0.28), (0.35,0.32), (0.44,0.25), and (0.38,0.21). These narrow, sample‐specific gamuts can capture subtle color variations more effectively than the wide gamut of conventional color reference charts (e.g., Macbeth ColorChecker) [64].

### 4.9 Design and Printing of Color Reference Chart

We designed the color reference chart using the CIE xy chromaticity and CIE L* (lightness) values within the Cl^−^ gamut, pH gamut, and OST gamut. The chart included 45 colors uniformly distributed within the pH gamut, 45 within the temperature gamut, and 44 within the Cl^−^ gamut. Additionally, 12 primary colors were incorporated to account for potential color outliers outside the three defined gamuts [64, 73]. A total of 146 sets of CIE xy chromaticity and CIE L* values, designed for the color reference chart, were then converted to their corresponding CIE XYZ values [72]. The resulting CIE XYZ values were mapped to sRGB for color representation in the finalized color reference chart. High‐fidelity reproduction of the designed chart was achieved using a professional‐grade inkjet printer (imagePROGRAF PRO‐1000, Canon) that utilizes 11 pigment‐based inks and a chroma optimizer (PFI‐1000 LUCIA PRO Ink, Canon). Accurate and precise calibration from digital input to printout was ensured by employing the International Color Consortium (ICC) profile and manufacturer‐specified genuine paper (Photo Paper Premium Fine Art Smooth, Canon).

### 4.10 Machine‐Readable Biomarker Quantification Via Color Correction

We developed algorithms to predict the three biomarkers (Cl^−^ concentration, pH, and OST) based on their corresponding color values. For model development, multiple samples were prepared for each sensor: six Cl^−^ samples (50, 100, 110, 135, 150, and 200 mm), five pH samples (pH 5–9, interval of 1), and ten temperature samples (31°C–40°C, interval of 1°C). Single‐shot photos of each sample alongside the designed color reference chart were captured using both an Android smartphone (Samsung Galaxy S21) and an iOS smartphone (Apple iPhone 12 Pro) in DNG format under white LED illumination at 5600 K (18‐Inch LED Ring Light Kit, NEEWER). The DNG format was used because it applies less color compression than JPEG, enabling more accurate and precise color correction with reduced computational burden. True CIE XYZ values under CIE illuminant E were recovered via polynomial color correction (Equation 6) using the color reference chart. For each sample, the final CIE XYZ values were obtained from measurements captured by both smartphone devices to account for device‐dependent imaging variations. Each biomarker was then modeled as a function of the true CIE XYZ values, enabling color‐based quantification. The regression model was trained using a subset of the collected color measurements and validated on independent image sets captured from both smartphone devices to assess robustness and minimize overfitting. Specifically, we derived a mathematical relationship between each sample parameter and its corresponding CIE XYZ values:

(7) $$b^{s}=i_{1\times 3}^{s}\;c_{3\times 1}^{s}$$

where $i_{1\times 3}^{s}=\left[x,y,z\right]\;$ is a vector of CIE XYZ values for each sample, *b_s_* is the corresponding biomarker value, and $c_{3\times 1}^{s}$ is a coefficient vector converting the CIE XYZ values to the biomarker value. Here, *s* = 1, 2, 3 denote the Cl^−^, pH, and OST sensors, and $b_{n\times 1}^{s}$ and $I_{n\times 3}^{s}$ were formed by stacking *b^s^* and $i_{1\times 3}^{s}$ from *n* = 6, 5, and 10 samples, respectively:

(8) $$b_{n\times 1}^{s}=I_{n\times 3}^{s}\;c_{3\times 1}^{s}$$

Multivariate polynomial regression was employed to expand $I_{n\times 3}^{s}$ to $I_{n\times e}^{s}$:

(9) $$b_{n\times 1}^{s}=I_{n\times e}^{s}\;c_{e\times 1}^{s}$$

where $I_{n\times e}^{s}$ can be expressed:

(10) $$I_{n\times e}^{s}=\left[\begin{array}{cccccc} x_{1} & y_{1} & z_{1} & \text{…} & x_{1}^{i}y_{1}^{j}z_{1}^{k} & \sqrt[{i+j+k}]{x_{1}^{i}y_{1}^{j}z_{1}^{k}}\\ \vdots & \vdots & \vdots & \vdots & \vdots & \vdots \\ x_{n} & y_{n} & z_{n} & \text{…} & x_{n}^{i}y_{n}^{j}z_{n}^{k} & \sqrt[{i+j+k}]{x_{n}^{i}y_{n}^{j}z_{n}^{k}} \end{array}\right]\;$$

where *i*, *j*, and *k* represent the degree of expansion. The coefficient vector $c_{e\times 1}^{s}$ in Equation 9 was obtained using the Moore‐Penrose pseudoinverse [71]. By substituting the color‐corrected CIE XYZ values of each sensor into Equation 9, the three biomarker values were computed for color‐based biomarker quantification.

### 4.11 In Vivo Evaluations in Rabbit and Canine Models

All animal experiments adhered to the Association for Research in Vision and Ophthalmology (ARVO) Statement for the Use of Animals in Ophthalmic and Vision Research and were approved by the Purdue Animal Care and Use Committee (protocol no. 2104002129). Six 6‐month‐old female New Zealand White rabbits (Envigo Global Services, Inc.) were used for device wearability and biocompatibility testing. For wearability assessments, sensors were integrated into 14.8 mm Zenlens scleral lenses. For the 24‐h biocompatibility study, sensing elements were instead embedded within 14.3 mm Acuvue Oasys soft contact lenses to better match rabbit corneal geometry and avoid scleral‐lens‐related irritation. Each rabbit received a sensor‐integrated soft lens in one eye and a bare soft lens in the contralateral eye, with assignment randomized and summarized in Table S3. Animals were housed under a 12‐h light/dark cycle.

Comprehensive ophthalmic evaluations including Schirmer tear testing (Merck Animal Health), tear film breakup time, fluorescein staining (Ful‐Glo; Akorn, Inc.), slit‐lamp biomicroscopy (SL‐17; Kowa, Inc.), and indirect ophthalmoscopy (Keeler, Inc.) were performed immediately before and after the 24‐h wear period. Any abnormalities were documented using a modified McDonald–Shadduck scoring system. Anterior segment OCT (Spectralis; Heidelberg Engineering, Inc.) was performed after approximately 1 h of wear to verify lens alignment and the position of the sensing components. To ensure lens retention during the 24‐h evaluation, partial temporary tarsorrhaphy was performed using 4‐0 nylon (Ethilon; Ethicon, Inc.). Rabbits were individually housed with unrestricted movement during the study. At study completion, rabbits were humanely euthanized, and ocular tissues including eyelids and adnexa were collected, fixed, paraffin‐embedded, sectioned, and stained with H&E for masked histopathological grading on a 0–3 semi‐quantitative scale (no lesions, minimal, mild, moderate, severe).

Functional assessment and in vivo colorimetric data collection were subsequently performed in a 15‐month‐old female beagle, selected for its close anatomical similarity to the human eye. A 16.0 mm sensor‐integrated Zenlens scleral lens was used to ensure proper vaulting and stable positioning during natural ambulatory activity. To capture physiologically relevant chromatic changes under real‐use conditions, sensor images were acquired under eight imaging settings comprising two smartphone devices (Samsung Galaxy S21 and Apple iPhone 12 Pro), two files format RAW (DNG) and JPEG), and two repeated acquisitions per device‐format combination. All images were processed using the previously described machine‐learning–based workflow to obtain corrected RGB values and produce quantitative electrolyte and temperature readouts.

### 4.12 Validation With Artificial Tear Fluids and Human Tear Samples

Artificial tear fluids with controlled chloride concentrations and pH values were prepared to evaluate the quantitative accuracy of the smart scleral lens sensors under tear‐relevant chemical conditions. Two formulations, ATF1 and ATF2, were prepared with target pH/Cl^−^ values of 7.0/110 and 8.0/150 mm. ATF1 contained 88 mm NaCl, 20 mm KCl, 1.0 mm CaCl_2_·2H_2_O, 5.0 mm sodium lactate, 0.14 mm glucose, 5.0 mm urea, and 50 µg mL^−^^1^ bovine serum albumin in deionized water. For ATF2, the NaCl concentration was increased to 128 mm, while all other components were kept the same. The total chloride concentration in each formulation was determined by the combined chloride contributions from NaCl, KCl, and CaCl_2_·2H_2_O. To avoid introducing additional chloride during pH adjustment, the pH was adjusted to 7.0 or 8.0 using NaOH or acetic acid, and the solutions were then brought to the final volume with deionized water and mixed thoroughly before use.

To validate the quantification algorithm, Cl^−^ and pH sensors were tested using ATF1 and ATF2 as reference samples with predefined Cl^−^ concentrations and pH values, while OST sensors were evaluated under controlled temperature conditions. Sensor images were acquired across six imaging settings generated by combining two smartphone devices (Samsung Galaxy S21 and Apple iPhone 12 Pro) with three LED illumination color temperatures of 3500, 4500, and 5500 K. All images were saved in RAW (DNG) format and analyzed using the previously described color correction and machine‐readable quantification workflow. The calibrated CIE XYZ values were used to predict Cl^−^ concentration, pH value, and OST, and the predicted values were compared with the corresponding reference values. Quantification accuracy and reproducibility were evaluated using root mean square error, mean absolute error, standard deviation, and coefficient of variation across imaging settings.

All tear collection procedures were performed by clinical research–trained personnel, in accordance with Good Clinical Practice (GCP) standards and relevant university regulations, and were approved by the Institutional Review Board (IU IRB protocol number: 2004308902). Informed consent was obtained from all participants prior to any study procedures. Clinical trial registration was not required for this pilot feasibility study; therefore, no clinical study registration number is applicable. There was no participant compensation. Human tear samples were collected from 10 adult volunteers using sterile, single‐use fine glass microcapillary tubes (10 µL capacity) following a non‐invasive protocol that did not involve corneal anesthesia or contact lens wear. Participants were seated in a reclining position and instructed to blink and avert their gaze during sample collection. Approximately 10 µL of tear fluid was drawn from the inferior tear meniscus of each eye, pooled, and transferred into low protein‐binding microcentrifuge tubes using a sterile plunger. All collection tubes were properly labeled, immediately placed on ice, and stored at –80°C until analysis. Concurrent with sample collection, the DEQ‐5 questionnaire and ocular surface staining were administered to assess the dry eye status of each subject. For ex vivo testing, sensors were immersed in thawed tear samples at room temperature for 1 h to allow equilibration with tear biomarkers. After incubation, the sensors were gently blotted to remove excess fluid and immediately imaged across four images comprising two smartphone devices (Samsung Galaxy S21 and Apple iPhone 12 Pro), saved in both RAW (DNG) and JPEG formats. The acquired RGB values were analyzed using the developed machine‐readable biomarker quantification algorithm based on color correction.

### 4.13 Statistical Analysis

For artificial tear fluid validation, predicted analyte values were compared against known reference values across six imaging settings (n = 6: two smartphones × three LED illumination color temperatures); accuracy was assessed using root mean square error (RMSE) and mean absolute error (MAE), while reproducibility across imaging settings was evaluated using the coefficient of variation (CV = standard deviation/mean). In the in vivo canine study, analyte values were predicted from eight imaging settings acquired from the same eye (n = 8: two smartphones × two file formats × two repeated measurements). For the human tear samples, each of the 10 participants was evaluated under four imaging conditions (n = 4: two smartphones × two file formats). Reproducibility in both datasets was assessed using CV. No data points were excluded as outliers, and all results are reported as mean ± standard deviation. Perceptual color differences between adjacent analyte concentrations (Figures S13–S15) were calculated using the CIEDE2000 formula in CIE LAB color space [74], implemented as a custom MATLAB function. Because the analysis characterizes measurement accuracy and reproducibility rather than comparing independent groups, no formal hypothesis testing (e.g., t‐test or ANOVA) was performed. All calculations were carried out using MATLAB R2021b (MathWorks).

## Author Contributions

Z.W., S.M.P., D.M., and S.A.L. contributed equally to this work. C.H.L. conceived the concept, planned the project, and supervised the research. Z.W., S.A.L., Y.L., T.Y., and C.H.L. designed, fabricated, and characterized the device. Z.W., S.A.P., Y.D., F.L., O.T.O., and G.C.S. designed and conducted the animal study. Z.W., S.M.P., and Y.L.K. designed and conducted the color study for biomarker quantification. D.M., G.C.S., and P.S.K. collected and analyzed the human tear sample. Z.W, S.M.P., and C.H.L. wrote the manuscript. All authors commented on the paper.

## Conflicts of Interest

The authors declare no Conflicts of Interest.

## Supporting information




**Supporting File 1**: advs77399‐sup‐0002‐SuppMat.docx.

**Supporting File 2**: advs77399‐sup‐0001‐S1.mp4.

## Data Availability

The data that support the findings of this study are available from the corresponding author upon reasonable request.
