# Supplementary material for: Electronics‐Free Visual Monitoring of Dry Eye Disease‐Related Tear Biomarkers Using Smart Scleral Lenses
Source: Adv Sci (Weinh). 2026 Aug 27:e77399. Online ahead of print. doi: 10.1002/advs.77399 (PMC13521194; doi:10.1002/advs.77399)
Supplement: Supplementary file 1 [file ADVS-9999-e77399-s001.docx]

Supporting Information for

**Electronics-Free Visual Monitoring of Dry Eye Disease-Related Tear Biomarkers Using Smart Scleral Lenses**

*Ziheng Wang^1‡^, Sang Mok Park^2‡^, Dawn Meyer^3‡^, Seul Ah Lee^4‡^, Yumin Dai^5^, Tianhao Yu^1^, Taewoong Park^4^, Feiyang Li^4^, Jason Gerard Jedlicka^3^, Tristan Michael Long^3^, Oluwabunmi T Oladele^6^, Gillian C. Shaw^7^, Shin Ae Park^6*^, Pete S. Kollbaum^3*^, Young L. Kim^2*^, Chi Hwan Lee^1,4,5,8*^*

^1^School of Mechanical Engineering, Purdue University, West Lafayette, IN 47907, USA. ^2^Department of Intelligent Systems Engineering, Indiana University, Bloomington, Indiana 47408, USA. ^3^School of Optometry, Indiana University, Bloomington, IN 47405, USA. ^4^Weldon School of Biomedical Engineering, Purdue University, West Lafayette, IN 47907, USA. ^5^School of Materials Engineering, Purdue University, West Lafayette, IN 47907, USA. ^6^Department of Veterinary Clinical Sciences, Purdue University, West Lafayette, IN 47907, USA. ^7^School of Veterinary Medicine, University of Wisconsin-Madison, Madison, WI 53706, USA. ^8^Elmore Family School of Electrical and Computer Engineering, Purdue University, West Lafayette, IN 47907, USA.

**The PDF file includes:**

Figures S1 to S17

Tables S1 to S3

Legends for movie S1

**Other Supplementary Material for this manuscript includes the following:**

Movies S1

**Figure S1.** **Schematic of the hydrogel-bead–based fabrication process for pH and Cl⁻ sensors.** Dry ion-exchange beads are hydrated and loaded with sensing reagents, followed by crosslinking to form stable hydrogel beads. The functionalized beads are subsequently embedded within a bulk hydrogel matrix to generate the integrated pH and chloride-responsive sensor film.

**Figure S2. RGB stability of the Cl⁻ sensor over a 12 h evaluation period.** RGB values of the Cl⁻ sensor were monitored at 0, 2, 4, 6, 8, and 12 h under fixed testing conditions. The red, green, and blue channels showed only minor variations over time, indicating stable colorimetric readout after initial color development (n = 3; mean ± s.d.).

**Figure S3.** **Optimization of bromothymol blue concentration for the pH sensor.** UV–Vis spectra of bromothymol blue at varying concentrations, showing systematic changes in the characteristic absorbance bands that define the sensor’s pH response.

**Figure S4. CIE xy chromaticity trajectory across the pH range.** Color responses of the pH sensor mapped onto the CIE xy chromaticity diagram, showing the systematic shift in perceived color with changing pH.

**Figure S5. Dye retention stability of the pH sensor after 8 h of immersion.**

**Figure S6. CIE xy chromaticity trajectory across the temperature range.** Color responses of the OST sensor mapped onto the CIE xy chromaticity diagram, showing the systematic shift in perceived color with changing temperature.

**Figure S7. Environmental robustness of the sensors**. RGB outputs remain stable during thermal cycling in solutions spanning pH 2–12 and Cl⁻ concentrations of 50–200 mM.

**Figure S8. SEM characterization of plain and dye embedded pHEMA–PEG hydrogel morphology in the dry state.** Plain pHEMA–PEG exhibits a wrinkled, continuous surface, whereas dye-embedded (bead-loaded) films show a fractured particulate morphology. (Scale bars:100 µm)

**Figure S9. Adhesion stability of the pHEMA–PEG hydrogel on the PMMA (Zenlens) substrate after PBS storage.**

**Figure S10. Environmental cycling tests demonstrating RGB stability of embedded sensors.** Repeated hydration–dehydration cycles produce minimal variation in RGB signals for the (A) pH and (B) Cl⁻ sensors. Similarly, thermal cycling between −20 °C and 50 °C preserves the RGB responses of the (C) pH and (D) Cl⁻ sensors, indicating strong environmental robustness of the hydrogel-embedded sensing elements (n = 5; mean ± s.d.).

**Figure S11. In vivo rabbit biocompatibility study of the sensor-embedded soft contact lens (SSCL).** (A) Anterior-segment OCT image obtained 1 h after the sensor integrated lens application demonstrates conformal lens placement with slight bending arising from mechanical mismatch between the hydrogel lens and the corneal surface (scale bar, 1 mm). (B) Clinical eye-exam images, including standard illumination and fluorescein staining, comparing bare lenses and SSCLs at baseline and after 24 h of continuous wear. Both conditions exhibit minimal ocular irritation, with no signs of corneal ulceration or epithelial compromise, confirming good short-term biocompatibility (scale bars: 1 cm).

**Figure S12.** **Sensor gamut characterization and construction of the lens-integrated color checker (color reference chart).** (A) CIE xy chromaticity gamuts for Cl⁻, pH, and OST derived from measured sensor colors. (B) Twelve Macbeth ColorChecker-equivalent primary colors outside the defined sensor gamuts for improving color correction accuracy of potential outlier colors. (C) Combined 146-color reference set (gamuts + primaries) converted to CIE XYZ and rendered as printable patches in the sRGB color space. (D) Final color chart layout designed for ICC-calibrated inkjet printing, used for smartphone color correction and quantification.

**Figure S13. Validation of machine-readable quantification of Cl⁻ concentration using color correction.** (A) Representative image of Cl⁻ sensors prepared at nine concentrations ranging from 0 to 200 mM, together with two artificial tear fluids (ATF1: 110 mM; ATF2: 150 mM). Each condition was prepared in triplicate (#1–#3). The color reference chart was placed alongside the sensors to enable color correction and recover setting-independent CIE XYZ values for algorithm development and validation. (B) Smartphone images acquired using different devices (Apple iPhone 12 Pro and Samsung Galaxy S21) and LED illumination color temperatures of 3500, 4500, and 5500 K in RAW (DNG) format. The measured RGB values varied substantially depending on the imaging setting. (C) Predicted Cl⁻ concentrations for each artificial tear sample across six imaging settings (n = 6, mean ± s.d.), showing a root mean square error (RMSE) of 4.42 mM and a mean absolute error (MAE) of 3.61 mM. Dashed lines indicate the ground-truth concentrations of the samples. (D) Coefficients of variation for each sample (n = 6), showing inter-setting variability below 0.03. (E) Perceptual color difference (ΔE, CIEDE2000) between adjacent Cl⁻ concentrations across the detection range of 0–200 mM, demonstrating sufficient color sensitivity for concentration-dependent quantification.

**Figure S14. Validation of machine-readable quantification of pH values using color correction.** (A) Representative image of pH sensors prepared at eleven pH values ranging from pH 2 to 12, together with two artificial tear fluids (ATF1: pH 7; ATF2: pH 8). Each condition was prepared in triplicate (#1–#3). The color reference chart was placed alongside the sensors to enable color correction and recover setting-independent CIE XYZ values for algorithm development and validation. (B) Smartphone images acquired under the same imaging conditions as in Figure S13B, using different devices (Apple iPhone 12 Pro and Samsung Galaxy S21) and LED illumination color temperatures of 3500, 4500, and 5500 K in RAW (DNG) format. The measured RGB values varied substantially depending on the imaging setting. (C) Predicted pH values for each artificial tear sample across six imaging settings (n = 6, mean ± s.d.), showing a root mean square error (RMSE) of 0.21 pH units and a mean absolute error (MAE) of 0.18 pH units. Dashed lines indicate the ground-truth pH values of the samples. (D) Coefficients of variation for each sample (n = 6), showing inter-setting variability below 0.03. (E) Perceptual color difference (ΔE, CIEDE2000) between adjacent pH values across the detection range of pH 2–12, demonstrating sufficient color sensitivity for pH-dependent quantification.

**Figure S15. Validation of machine-readable quantification of ocular surface temperature (OST) using color correction.** (A) Representative images of the OST sensor acquired at controlled temperatures from 31 to 40 °C at 1 °C intervals, together with two artificial tear samples. Each temperature condition was tested in triplicate (#1–#3). The color reference chart was placed alongside the sensor to enable color correction and recover setting-independent CIE XYZ values for algorithm development and validation. (B) Smartphone images acquired using different devices (Apple iPhone 12 Pro and Samsung Galaxy S21) and LED illumination color temperatures of 3500, 4500, and 5500 K in RAW (DNG) format. The measured RGB values varied substantially depending on the imaging setting. (C) Predicted OST values across six imaging settings (n = 6, mean ± s.d.) at three representative temperatures of 33, 36, and 39 °C, showing a root mean square error (RMSE) of 0.92 °C and a mean absolute error (MAE) of 0.76 °C. Dashed lines indicate the ground-truth temperatures for each condition. (D) Coefficients of variation for measurements obtained at 33, 36, and 39 °C (n = 6 per temperature), showing inter-setting variability below 0.04. (E) Perceptual color difference (ΔE, CIEDE2000) between adjacent OST values across the detection range of 31–40 °C, demonstrating sufficient color sensitivity for temperature-dependent quantification.

**Figure S16. Selectivity of the (A) Cl⁻, (B) pH, and (C) OST sensors against tear-relevant interferents.** The OST sensor was evaluated at room temperature for all tested conditions. n = 3; data are presented as mean ± s.d.

**Figure S17. Additional corneal and conjunctival fluorescein staining images.**

| **Biomarker** | **Clinical Importance in DED** | **Typical Range** | **Observed in DED** | **References** |
| --- | --- | --- | --- | --- |
| **Cl⁻ concentration** | - Central pathogenic factor in DED. - Elevated osmolarity (reflecting higher tear Cl⁻) triggers cellular stress, inflammatory cascades, and epithelial apoptosis. | ~122.86 ± 7.12 mM | Cl⁻ concentration increases by ~5–10% in DED. | (51*, 55*) |
| **pH** | - Reflects ocular surface inflammation and tear film imbalance. - pH shifts (often toward alkalinity) can indicate disrupted epithelial homeostasis and mucin instability. | 6.5–7.6 | Slight alkalization reported in moderate-to-severe evaporative DED (exact DED-associated pH ranges not widely standardized) | (*52, 53, 56*) |
| **Ocular surface temperature (OST)** | - Reflects tear film evaporative stability and inflammation. - DED (evaporative subtypes) is associated with dysregulated temperature dynamics (e.g. accelerated cooling rate) due to increased tear evaporation. | Modest cooling (~0.2–0.3 °C drop post blinking) | Faster surface cooling (~0.78 ± 0.30 °C drop post blinking) | (*54*) |

**Table S1.** Clinical significance of the selected biomarkers.

| **Species** | **Typical concentration in human tears** | **Interaction with sensing chemistry** | **Expected interference** | **References** |
| --- | --- | --- | --- | --- |
| **Cl⁻** | ~122.86 ± 7.12 mM | Reacts with silver chloranilate to form AgCl | Primary sensing target | (*51, 55*) |
| **Br⁻** | ~0.06 ± 0.01 mM | Can theoretically form AgBr with Ag⁺ | Negligible due to very low concentration | *(56)* |
| **I⁻** | Not reported / trace | Can theoretically form AgI with Ag⁺ | Negligible (not detected in tear composition studies) | *(57)* |
| **Na⁺** | ~132 mM | No reaction with silver chloranilate | None | *(57)* |
| **K⁺** | ~24 mM | No reaction with silver chloranilate | None | *(57)* |
| **Lactate** | ~0.5–3 mM | Does not react with Ag⁺ | None | *(58)* |
| **Urea** | ~4.5–7 mM | No precipitation or colorimetric reaction | None | *(59)* |
| **Glucose** | ~0.1–0.6 mM | No interaction with sensing reagent | None | *(60)* |
| **Proteins** | ~4.34± 1.91 μg/μL | No direct reaction with sensing chemistry | Minimal | *(61)* |

**Table S2.** Major tear components and their potential interaction with the Cl⁻ sensing chemistry.

| **Animal ID** | **Sensor Eye** | **Bare Lens Control** | **Right Eye (OD)** | **Left Eye (OS)** |
| --- | --- | --- | --- | --- |
| **1** | OS | OD | 0.5 | 0.5 |
| **2** | OS | OD | 0.5 | 0.75 |
| **3** | OD | OS | 0.5 | 0.5 |
| **4** | OS | OD | 0.5 | 0.5 |
| **5** | OD | OS | 0.5 | 0.5 |
| **6** | OS | OD | 0.75 | 0.75 |

Grades for assessment of inflammation:

0.5 = minimal, 1= mild, 2= moderate, 3 = severe

**Table S3. Quantitative histopathologic inflammation scores of the rabbit cornea and conjunctiva.** Histopathologic evaluation of corneal and conjunctival tissues was performed using established grading criteria, permitting quantitative comparison of inflammatory responses following lens or sensor wear.

**Movie S1.**

In vivo smart scleral lens evaluation in dog eye.
